# Biofilms in Diabetic Foot Ulcers: Impact, Risk Factors and Control Strategies

**DOI:** 10.3390/ijms22158278

**Published:** 2021-07-31

**Authors:** Ana C. Afonso, Diana Oliveira, Maria José Saavedra, Anabela Borges, Manuel Simões

**Affiliations:** 1 LEPABE—Laboratory for Process Engineering, Environment, Biotechnology and Energy, Faculty of Engineering, University of Porto, Rua Dr. Roberto Frias, s/n, 4200-465 Porto, Portugal; up202010573@edu.fe.up.pt (A.C.A.); dianarosalopesoliveira@gmail.com (D.O.); apborges@fe.up.pt (A.B.); 2 CITAB—Centre for the Research and Technology for Agro-Environment and Biological Sciences, University of Trás-os-Montes e Alto Douro, 5001-801 Vila Real, Portugal; saavedra@utad.pt; 3 CEB—Centre of Biological Engineering, Campus de Gualtar, University of Minho, 4710-057 Braga, Portugal; 4 CIQUP, Department of Chemistry and Biochemistry, Faculty of Sciences, University of Porto, Rua do Campo Alegre, s/n, 4169-007 Porto, Portugal; 5 Department of Veterinary Sciences, School of Agrarian and Veterinary Sciences, University of Trás-os-Montes e Alto Douro, 5001-801 Vila Real, Portugal

**Keywords:** biofilms, chronic wounds, combination therapy, multidrug resistance, pathophysiology, polymicrobial aetiology

## Abstract

Diabetic foot ulcers (DFUs) are a serious complication from diabetes *mellitus*, with a huge economic, social and psychological impact on the patients’ life. One of the main reasons why DFUs are so difficult to heal is related to the presence of biofilms. Biofilms promote wound inflammation and a remarkable lack of response to host defences/treatment options, which can lead to disease progression and chronicity. In fact, appropriate treatment for the elimination of these microbial communities can prevent the disease evolution and, in some cases, even avoid more serious outcomes, such as amputation or death. However, the detection of biofilm-associated DFUs is difficult due to the lack of methods for diagnostics in clinical settings. In this review, the current knowledge on the involvement of biofilms in DFUs is discussed, as well as how the surrounding environment influences biofilm formation and regulation, along with its clinical implications. A special focus is also given to biofilm-associated DFU diagnosis and therapeutic strategies. An overview on promising alternative therapeutics is provided and an algorithm considering biofilm detection and treatment is proposed.

## 1. Introduction

Diabetes *mellitus* (DM) is one of the most prevalent endocrine diseases worldwide, characterised by an increase in blood glucose levels caused by defective insulin secretion, action or both [1]. Officially, diabetes is classified into three major groups, namely, Type 1, Type 2 and gestational [2]. Type 1 DM or insulin-dependent DM (IDDM) is the result of a failure of the body to produce insulin, due to destruction of pancreatic β-cells [3]. This type represents only 5–10% of all diabetes cases and is mostly associated to genetic factors [2]. Type 2 DM, also known as non-insulin dependent DM (NIDDM), represents the most common type comprising 85% of all cases [2]. This Type results from insulin resistance or insufficient insulin production, essentially associated with multihormonal disorders [4]. Gestational DM (GDM) is mainly caused by a blockage of insulin action by the pregnancy hormones, causing insulin resistance and hyperglycaemia [5]. Women with a history of GDM are more likely to develop Type 2 diabetes later in their lives [5].

The global prevalence of diabetes increases every year. In fact, the number of people with diabetes between 1980 and 2014 quadrupled, from 108 million to 422 million, and the numbers continue to escalate [6]. This increase is so fast that diabetes is now considered by the World Health Organization (WHO) as a global fast-growing epidemic [6]. According to epidemiological studies, if the current trend continues, by 2045, 700 million adults will suffer from the disease, which represents a 51% increase [7,8]. In addition to this global public health threat, there is a huge economic burden associated. The related annual global health expenditure will rise 11% from USD 760 billion in 2019 to USD 845 billion by 2045 [8].

In addition to the economic impact associated with this disease, there are other multifaceted complications that account for more than 50% of these direct costs, such as nephropathy, neuropathy, retinopathy, atherosclerosis and foot ulcers [9,10,11,12,13]. These secondary pathophysiological outcomes are a result of a deficiency in the vascular system, causing inefficient circulation [14]. Among all of these diabetes complications, foot ulcers are at higher risk to occur, and it is estimated that 20% of hospital admissions among DM patients result from diabetic foot ulcers (DFUs) [14,15]. DFUs can lead to infection, gangrene, amputation, and if proper treatment is not provided, can even cause death [14,15]. In fact, once a DFU is developed there is a greater risk of amputation, and it is estimated that 50–70% of all lower limb amputations (LLAs) are due to DFUs [14]. It is predicted that in the general population (≥45 years), the incidence of vascular LLA in the diabetic is eight times higher than in non-diabetic individuals, and when it comes to the age group ≥ 85 years, the incidence in men increases to 15 times higher and 12 times higher in women than the mean incidence rates of all population groups [16]. Unfortunately, the COVID-19 outbreak has had a negative impact on healthcare delivery to patients with DFUs [17,18]. In fact, a study in Naples reported that patients with diabetes admitted to a Tertiary Care Center for DFU management had a threefold risk of amputation compared to 2019 numbers [19]. LLA has a greater negative impact on the patient’s quality of life than any other complication of diabetes, such as renal failure or blindness, with depression and anxiety highly associated [12,20]. Regarding the considerable morbidity associated, it has been reported that every 30 s, one leg is amputated worldwide due to DFUs [14].

DFUs can be colonised by pathogenic bacteria and infection is favoured by the immunological deficiencies related to diabetes [21,22]. The pathogens involved in these infections vary from aerobic to anaerobic species, which may include *Staphylococcus* spp., *Streptococcus* spp., Proteobacteria, *Pseudomonas aeruginosa* and coliform bacteria [14]. In DFUs, the different microorganisms can exist either in planktonic or sessile state [23]. When bacteria form biofilms, the cells are embedded in a self-produced polymeric matrix, which confers them protection from the host’s immune system and from antibiotics [24]. As a consequence, biofilms in DFUs may be responsible for the delayed healing and consequent infection chronicity [25,26,27], despite systemic antibiotic treatment [28].

This review aims to provide insights to DFU causes, predisposition factors, and the global impact on the economy and society. Furthermore, measures usually applied for the prevention of new/recurrent DFUs, and the management and treatment of DFU infections are reviewed. Emphasis will be given to the biofilm’s contribution to DFU infection exacerbation and persistence. The main tools used for the diagnosis and treatment of biofilm-associated DFUs are also reviewed.

## 2. Epidemiology and Risk Factors of Diabetic Foot Ulcers

The diabetic foot is a major medical, social and economic problem, affecting 40 to 60 million people globally [29]. The main risk factors for diabetic foot ulceration are older age, male sex, Type 2 diabetes, lower body mass index, longer diabetic duration, hypertension, diabetic retinopathy and a history of smoking [9,30]. DFUs, especially chronic ulcers, can lead to amputations which can cause a significant decrease in life quality and an increase in early mortality [8]. The five-year relative mortality after a DFU is 48%, which is a higher value than that of many types of cancers [31]. However, the reported frequency of ulceration varies considerably. For example, the prevalence of active DFUs ranges from 3% in Oceania to 13% in North America, with a global mean prevalence of 6.3% [30]. This difference is even greater when comparing DFU prevalence among countries [30]. For example, Belgium has the highest prevalence of DFUs with 16.6%, whereas Australia presents the lowest value: 1.5% [30]. These disparities are justified by some differences in the healthcare systems. For instance, in Belgium, the state supports the treatment costs, but not the expenses related to preventive treatments, whereas in Australia, fewer than 50% of diabetic patients have regular foot examinations [32,33].

## 3. Social and Economic Burden of Diabetic Foot Ulcers

DFUs have a significant impact from both the patient perspectives as well as from a medical and economic standpoint. Diabetic patients with foot ulcers bear health expenditures five times higher than those without it [34]. In the United States, around USD 17 billion are spent annually for diabetic foot care, while the European Commission (EC) estimates that these costs are approximately EUR 2.5 billion per year [12,35]. Intangible costs, those which cannot be measured, also have a significant impact on patient’s lives. These costs include anxiety, frustration, discomfort, pain, loss of independence, and others which arise, for example, due to concerns about managing the condition, fear of future complications and impact on the quality of life [8]. However, early detection and improved management of diabetes complications will benefit not only the quality of life of individuals with diabetes, but also the health economy in general [36].

## 4. Etiopathogenesis of Diabetic Foot Ulcers

The aetiology of DFUs is multifaceted; it results from the simultaneous action of multiple contributing causes. In addition, because metabolic mechanisms are impaired in DM, the risk of infection and/or poor wound healing is high [37,38]. Monteiro-Soares et al. performed a systematic review on the association between independent variables and DFUs [38]. The authors included 71 studies and evaluated the association between DFUs and more than 100 independent variables. Diabetic neuropathy, peripheral arterial disease, foot deformity and previous diabetic foot ulceration or lower extremity amputation were consistently associated with diabetic foot ulceration development [38]. Of these, peripheral neuropathy (neuropathic ulcer), peripheral arterial disease (ischemic ulcer), or both (neuro-ischemic ulcer) were considered the major underlying causes [38]. Moreover, a combination of these primary risk factors and other causal factors may be involved [39,40]. Other factors such as poor glycaemic control (which progressively leads to peripheral neuropathies), poor hygiene habits, lack of regular surveillance of skin integrity, non-premature detection of injuries and prolonged pressure in specific areas, can also be of relevance and, if controlled in a timely manner, can prevent wounding or wound progression. However, these are mainly under the patients’ control [9,41].

### 4.1. Peripheral Neuropathy

Diabetic peripheral neuropathy is characterised by the impairment of normal activities of the nerves, causing ulceration due to trauma or excessive pressure on a deformed foot without protective sensitivity [42]. The prevalence of diabetic peripheral neuropathy ranges from 16% to as high as 66% [43]. In neuropathy, hyperglycaemia increases the action of the enzymes aldose reductase and sorbitol dehydrogenase, resulting in the conversion of intracellular glucose into sorbitol and fructose. As these products accumulate, myoinositol synthesis of nerve cells is reduced, affecting nerve conduction [44]. The damage of innervations leads to an imbalance between flexion and extension of the affected foot, resulting in anatomical deformities with abnormal bony prominences and pressure points which cause gradual ruptures of the skin and ulceration [44]. Diabetic neuropathy is manifested in motor, autonomic and sensory components of the nervous system [40].

Motor neuropathy causes dysfunctions in leg muscles, resulting in the protrusion of abnormal bones, alteration of the normal foot architecture and foot deformities, such as hammertoes and hallux rigidus [45]. Damage to the innervation of leg muscles causes an imbalance between flexion and leg extension, resulting in deformity and change in pressure points [46]. Progressively, it will lead to skin damage that develops into ulcers. Autonomic neuropathy is manifested by the interruption of microvascular blood flow and sudomotor function that results in dry skin without sweating, which, in turn, triggers fissures and skin crust and makes the foot vulnerable to minimal trauma [47]. Sensory neuropathy presents a loss of protective sensation, resulting in susceptibility to physical and thermal trauma as well as increased risk to developing foot ulcers [48]. In short, diabetic peripheral neuropathy results not only in the loss of the proprioception of foot position, but also in the loss of pain and pressure sensation. This sensory deficit may lead to the presence of an unknown ulcer [44]. Once the protective layer of the skin is damaged, tissues are exposed to bacterial colonisation and are thus more prone to form complex microbial communities such as biofilms [42].

### 4.2. Peripheral Arterial Disease

Peripheral arterial disease contributes to the development of foot ulcers in up to 50% of cases [49,50]. In peripheral arterial disease, hyperglycaemia causes vascular endothelial dysfunction as well as decreased vasodilator production by the endothelium, which results in blood vessel constriction [51]. Hyperglycaemia in diabetes increases thromboxane A2, a vasoconstrictor with prothrombotic properties that results in an increased risk of hypercoagulability [52]. Hypertension and dyslipidaemia also contribute to the occurrence of peripheral arterial disease [53]. These factors cause occlusive arterial disease, which results in ischaemia of the lower extremities and increases the risk of developing ulcers [44]. In this way, the formed ulcers can be easily infected, evolve into gangrene, and end up with a lower leg amputation (below the knee) [54].

## 5. Preventive Measures for Diabetic Foot Ulcers

According to International Working Group on the Diabetic Foot (IWGDF) guidelines (2019), some key points can help to prevent DFUs: (i) identify the at-risk foot; (ii) regular inspection and examination of the at-risk foot; (iii) educate the patient and the patient’s family; (iv) ensure the wearing of appropriate footwear; and (v) treat risk factors for ulceration [55]. These key points should be address by a specialised team of healthcare professionals [55]. The early detection and proper management of DFUs will have benefits, not only for people with diabetes, but also for the wider health economy [8].

In a first instance, a regular examination of the feet should be performed. In the absence of symptoms, the risk of foot ulceration is low, but this cannot be neglected, and an annual examination must be performed [55]. Knowledge of foot self-care practices and the recognition of signs of ulceration should be taught to people with diabetes [55]. Minor changes in behaviour, such as shoe replacements, are also important. Wearing inappropriate shoes or walking barefoot are the main causes of foot trauma that lead to foot ulceration in diabetics [38]. It is recommended to use tailored shoes to suit any change in the foot structure or foot biomechanics [56]. Treating any modifiable risk factor or pre-ulcerative sign is also recommended. This includes removing corns; protecting the bubbles or draining them if necessary; treating ingrown or thickened nails; and prescribing appropriate therapeutics for fungal infections. The treatment must be continued until these abnormalities disappear, without recurrence. If these preventive measures fail, surgical intervention should be considered [55].

## 6. Management and Treatment of Diabetic Foot Ulcers

Predicting the appearance of chronic wounds such as foot ulcers in diabetic patients is almost impossible. Therefore, when preventive measures fail and the development of DFUs occurs, appropriate treatment should start immediately [57]. The primary purpose of basic wound care involves keeping the wound base free of nonviable tissue, facilitating a measurable and meaningful wound size reduction, which ultimately will lead to its healing [58]. The healing of DFUs requires not only the control of the infection, following the universal principles of hygiene and proper dressing of the ulcer, but also the adequate restoration of the vascularity and other extra care regarding the metabolic regulation of glycaemia and lipids, the cessation of smoking and weight reduction [59]. Indeed, ulceration usually occurs in the high-pressure bearing area of the foot [60]. Patients with diabetes have higher peak plantar pressures, along with repetitive pedal stress caused by bone and structural abnormalities of the foot in the presence of neuropathy and peripheral arterial disease [61,62]. As a result, the redistribution of constant plantar pressure is of utmost importance in managing a DFU in remission [63]. In DFU treatment, the regular wound maintenance implies several steps, including offloading, debridement, antimicrobial therapy and dressing.

Offloading is the most important element for the management of neuropathic ulcers. It is an intervention characterised by the relief of plantar pressure, because it avoids high-pressure load in the ulcerated area while still allowing the patient to walk [64]. This pressure redistribution prevents any trauma to the tissue and facilitates wound healing [65,66]. The most effective offloading device is total contact casting (TCC) [48]. This device is designed and moulded carefully to the shape of the foot, which thereby forces compliance, reduces activity levels, and consequently, improves wound healing [67]. Some evidence suggests that TCC boosts the reduction in wound pressure, prompting healing rates between 73% and 100% [68,69,70,71,72].

Another critical step in DFU management which should be performed before the application of any wound closure treatment is debridement [73]. Debridement is a therapy that involves the removal of nonviable tissue and debris at the wound margins [74]. The reasoning for serial debridement is to activate senescent cells, stimulate the release of growth factors, remove inflammatory factors, and reduce bioburden [75]. It also helps in the elimination of the base of abnormal injury, callus (epidermal hyperkeratosis), necrotic tissue and bacterial elements that inhibit wound healing [76]. There are different debridement methods, namely, surgical (for sharp debridement), biosurgical (use of maggots), autolytic (use of hydrogels, hydrocolloids and transparent films), biochemical (use of enzyme preparations), chemical (use of antiseptics, polysaccharide beads and pastes) and mechanical (use of hydrodebridement) [77]. Among all these debridement methods, the one considered the gold standard, and thus contributing the most to wound healing, is the surgical or sharp debridement method [48]. This technique is a rapid and effective way to remove dead tissue. Ideally, this procedure should be performed in combination with other treatment approaches, such as growth factors or cell-based therapies.

Typically, due to the high incidence rate of DFU infections, it is necessary to combine the conventional methodologies described above with an appropriate antibiotic regimen. This initial antibiotic treatment is generally empirical to cover the most commonly infecting microorganisms. However, this routine should be revised according to the severity of the infection or to the available microbiological data [78,79]. In fact, this information should be a subject of detailed study through microbiological cultures in order to determine the causative pathogens, and most importantly, their susceptibility to antibiotics [78,79]. In fact, there are some recommendations proposed by the Infectious Diseases Society of America (IDSA) for empirical antibiotic regimens considering the associated pathogen and the clinical severity of the infection (Table 1). According to these recommendations, narrow-spectrum oral antibiotics must be administered for mild infections, oral or parenteral antibiotics for moderate infections, and broad-spectrum parenteral antibiotics for severe infections. The empiric regimen should always include antibiotics against strains of *Staphylococcus* and *Streptococcus* species and, in some specific situations, include antibiotics against Gram-negative rods, methicillin-resistant *Staphylococcus aureus* (MRSA), *Pseudomonas* spp., multidrug-resistant (MDR) bacteria and anaerobes [80]. Empirical antibiotic therapy can be continued or adjusted according to the culture results and the patient’s clinical response. If a clinical improvement is observed and there is no serious infection, empirical treatment can be continued. This procedure is adopted even if the antibiotic susceptibility results show that some or all isolated microorganisms are resistant to the prescribed agents [81]. Likewise, if it is a multispecies culture, it may be sufficient to treat only the likeliest pathogens (e.g., *S. aureus*, *Streptococcus* species and Enterobacteriaceae) [82,83]. However, if the infection does not respond, the antibiotics initially selected should be replaced with alternatives with a broad-spectrum of action. If the infection worsens further, despite proper antimicrobial therapy, other options should be considered, such as surgery or the use of advanced therapies.

A major breakthrough for DFU management has been the design of novel dressings. Ideally, dressings should alleviate symptoms, provide wound protection and help wound healing [84]. Dressings have been engineered to guarantee a moist environment (favourable for healing), while also controlling microbial growth, allowing gaseous exchange, and thermally insulating the wound [85,86]. When choosing the dressing for an infected DFU, some factors must be taken into consideration: it should be comfortable and adequate for the patient as well as help to alleviate or at least not worsen the pain, especially during dressing changes; it should aid in the management of the infection itself; allow the observation of the wound; provide mechanical protection and conformability; and should be cost-effective [87]. There are a wide variety of dressings commercially available for the treatment of DFUs, and new products are frequently being developed and released, each one aimed at different aspects of healing [85,88]. In Table 2, different classes of dressings and their advantages/disadvantages are presented.

Despite some success in the management of DFUs using conventional methods, there are still some cases without desired outcomes. For these situations, advanced therapies have been developed either alone or in combination with these conventional techniques to increase the healing rates, reduce the treatment time, and decrease the probability of amputation. Among the most advanced therapies used are hyperbaric oxygen therapy (HBOT), negative pressure wound therapy (NPWT), bioengineering skin (BES) substitutes and growth factors.

HBOT is a methodology that involves the daily intermittent administration of 100% oxygen to the wound through an airtight vessel at a pressure higher than atmospheric (2–3 atm) [89,90]. As a result, this technique will increase the concentration of dissolved oxygen in plasma; therefore, this will lead to a rise in the diffusion of oxygen into tissues [89]. The exact mechanism of action of HBOT is not yet fully understood; however, there are some reports suggesting that improved healing is caused by the better replication of fibroblasts, endothelial cells and keratinocytes in an oxygen-rich environment, and the more effective killing of bacteria by leukocytes [48,91]. Despite the reports of an increased healing rate, this technique is not recommended as an adjuvant therapy. This is a technique which does not substitute the conventional treatment and, in addition, has huge associated costs (between USD 50,000 and 200,000) [15]. Furthermore, and based on a systematic review conducted by the National Institute for Health and Clinical Excellence (NICE) Guidelines Development Group in the United Kingdom, it was concluded that the data available were not robust enough to demonstrate that this technique is indeed cost-effective [92].

NPWT is considered to be an innovative treatment for DFUs. It is based on the use of a specific pump to create a localised sub-atmospheric pressure that will help the wound to heal [48]. Within this process, a closed system is created by using polyurethane or polyvinyl alcohol foam dressings, which are fitted to the wound surface, and an adhesive drape for coverage [15,48]. The suction generated by the pump will allow the collection of wound fluids, discharge and exudate [15,46]. This therapy has been shown to enhance oedema and exudate removal, to reduce bacterial colonisation, and to improve the blood flow [93]. There is promising evidence of the success of NPWT in patients with DFUs; however, it appears only to be effective as a postsurgical treatment [15]. NPWT should only be performed after debridement, and continued until the formation of granulated tissue [48]. Studies have suggested that NPWT significantly reduces healing times and increases the healing rate [94,95].

Another relatively recent therapeutic method used to manage DFUs involves the use of BES substitutes [96,97,98]. This method comprises the replacement of the degraded milieu of extracellular matrix by a new matrix with cellular/acellular components [15]. These BES substitutes can be classified into three major groups, allogenic cell-containing, autologous cell-containing and acellular matrices [48]. The first two include matrices containing living cells, such as keratinocytes and fibroblasts, whereas the latter comprises the release of growth factors [48]. Basically, this product is based on scaffolds that are seeded with human cells or growth factors which are then cultured in vitro. This culture will function as a delivery system that allows the cell-secreted matrix and growth factors to accumulate in the scaffold and actively be secreted at the wound bed [96,98]. Currently, there are some BES products which have been approved, namely, AlloDerm^®^ (LifeCell Corporation, Branchburg, NJ, USA); Apligraf^®^ (Graftskin, Organogenesis Inc., Canton, MA, USA); Dermagraft^®^ (Advanced Biohealing Westport, CT, USA); GRAFTJACKET^®^ (Wright Medical Group Inc., Arlington, TN, USA); Hyalograft^®^ 3D (Fidia Advanced BioPolymers, Abano Terme, Italy); Laserskin^®^ (Fidia Advanced BioPolymers, Abano Terme, Italy); OASIS^®^ (Cook Biotech, West Lafayette, IN, USA); OrCell^®^ (Ortec International Inc., New York, NY, USA); and TranCell^®^ (CellTran Ltd., Sheffield, UK) [48]. Despite all the advantages of BES mentioned above, it cannot be used alone for the treatment of DFUs due to wound-specific characteristics that may affect transplantation, as is the case with peripheral ischaemia [15]. Therefore, for this procedure, preparation of the wound bed including surgical revascularisation, decompression and infection control are necessary steps [15]. Taking everything into consideration, it appears evident that this therapy may lead to elevated long-term costs and treatment periods.

The use of growth factors is also reported to benefit DFU treatment, particularly of non-infected wounds [48]. This therapy includes the use of growth factors such as fibroblast growth factor, vascular endothelial growth factor, platelet-derived growth factor (PDGF), insulin-like growth factor (IGF1, IGF2), epidermal growth factor and transforming growth factor beta [15,99]. Among these growth factors, the only one approved by FDA is recombinant human PDGF, also known as becaplermin or Regranex [15,48]. This product is a hydrogel containing human platelet-derived growth factors which, when used in combination with debridement, have been demonstrated to increase ulcer healing [100,101,102]. Initially, this gel produced some positive results; however, it has been associated with an increased incidence of cancer, especially in patients treated with high doses [103]. Therefore, the FDA published a warning note regarding the use of high doses of becaplermin.

Failures or delays of DFU healing in patients that received optimal standards of care and still have evident clinical infection can be explained by the presence of a biofilm [104]. Infections related to biofilm occurrence are usually different from those promoted by the same microorganisms in a planktonic state; therefore, the therapeutic schemes are usually ineffective, resulting in non-healing chronic wounds [105].

## 7. The Role of Biofilms in Diabetic Foot Ulcers

The skin microbiota is essential for the regulation of the host immune system, the maintenance of epithelial barrier function, and protection against invading pathogenic microorganisms [106]. After injury, skin microbiota and pathogenic species may colonise the wound and proliferate [107]. In fact, studies have demonstrated that species isolated from chronic wounds are commensal on healthy skin [41,108,109,110]. For example, Park et al. analysed the microbiota of diabetic foot wound (DFW) tissue compared with normal foot skin in the same patients [110]. The authors found a myriad of *Actinobacteria, Staphylococcus, Corynebacterium*, and *Propionibacterium* species in normal samples compared to the infected tissue, and a relative abundance of anaerobes (*Bacteroides* and *Enterococcus*) and *Pseudomonas* species in wound tissues. Anaerobes in wounds can impair wound healing and increase the severity of infection [111]. These results are in agreement with other studies [108,109,110], suggesting that DFUs are associated with polymicrobial colonisation [112,113], containing more anaerobic bacteria than other wounds [114]. In addition to the expression of several virulence factors, polymicrobial communities can be organised into biofilms, responsible for DFU chronicity [112,115].

Biofilms are complex communities, consisting of microbial cells embedded in a self-produced matrix of extracellular polymeric substance (EPS). This matrix is composed of proteins, lipids, nucleic acids, polysaccharides and other components which confer the ability of bacteria to adhere to biotic or abiotic surfaces [115,116]. Biofilms are ubiquitous in nature and have a considerable impact on the healthcare sector. It is estimated that around 65% of all bacterial infections in humans are associated with biofilms [73]. Actually, they have been associated with several different illnesses, such as nephrolithiasis, endocarditis, cystic fibrosis, oral infections, and infections associated with indwelling devices [117]. In addition, biofilms exist in both chronic and acute wound infections [118], offering some ecological and physiological advantages to bacteria [119]. In fact, biofilms provide a physical protection to bacteria, which prevents antimicrobial agents (disinfectants and antibiotics) from penetrating the complex structure of biofilms, decreasing the concentration of antimicrobials acting against the embedded cells [120]. Additionally, due to close cell–cell contact in biofilms, horizontal gene transfer of antibiotic-resistant genes is facilitated [121]. In addition, due to the characteristics mentioned above, during a biofilm infection, host immune responses generated are largely ineffective, which leads to recurrent or chronic infections [122,123].

### 7.1. Biofilm-Associated Diabetic Foot Ulcer Infections

Biofilms have a crucial role in diabetic foot infections because the colonising bacteria act synergistically, creating a symbiotic environment favourable for the infection progression and, therefore, for the generation of a chronic wound [23]. Bacteria present in chronic wounds find the environment ideal for the formation of these polymicrobial sessile communities [105]. Furthermore, necrotic tissue and debris present in DFUs allow bacterial adhesion and biofilm formation [105]. In contrast to planktonic cells, biofilms are almost impossible to treat and eradicate [112,124,125]. In fact, biofilm formation is considered one of the reasons why wound infections treatment with conventional antimicrobial agent fails [116,126]. The presence of biofilms has been found in approximately 60–80% of chronic wounds and 6% of acute wounds, which strongly implies their contribution to hinder wound healing [127]. Bacteria in planktonic state express virulence factors that favour acute infections, whereas the sessile state is related to chronic infections [115,128].

A chronic inflammatory response is generated in an attempt to clean the biofilm from the wound. Thus, there is an accumulation of neutrophils and macrophages around biofilms, which secrete high levels of reactive oxygen species (ROS) and proteases [129]. Proteases can help to break down the attachment between the biofilm and the tissue [129]. However, ROS and proteases can impair healing by damaging normal or regenerating tissues, and interfering with immune system proteins and cells [130]. Neutrophils play an important role in the wound healing process by removing bacteria, foreign material, and necrotic tissue, and releasing cytokines to promote revascularisation and fibrosis [105]. On the other hand, the prolonged existence of neutrophils can lead to the release of inflammatory factors, ROS and proteinases that degrade the extracellular matrix and proteins involved in the healing cascade, causing a delay in wound healing [131]. Matrix metalloproteinases (MMPs) are an example of proteinases that play a major role in wound healing [132]. MMP levels decrease through the normal wound-healing process, whereas the exaggerated inflammatory phase in chronic wounds leads to an excess of proteases and inflammatory cytokines released by neutrophils and macrophages [133,134,135]. Lobman et al. found that the levels of some MMPs (MMP-1, MMP-8, MMP-9 and activated MMP-2) were significantly higher in DFUs [136]. In contrast, the level of the tissue inhibitor of MMP (TIMP), TIMP-2, was significantly lower compared to acute wounds from non-diabetic patients. These results suggest that impaired healing in DFUs can be caused by the imbalance between an excess of MMPs and a decrease in TIMPs [134]. Recently, Chang and Nguyen observed different effects on the healing process using different selective inhibitors for MMP-8 and MMP-9 proteinases, identified from the wounds of diabetic mice and humans [137]. Their investigation showed that infection increases MMP-9 levels, incrementing inflammation and decreasing angiogenesis, whereas MMP-8 is beneficial for wound repair. That said, the authors suggest that the best strategy for treating DFUs is to selectively inhibit the MMP-9 proteinase without affecting the beneficial MMP-8 [137]. Research is needed on the influence of MMPs and biofilms on the healing of chronic wounds. However, evidence suggests that with treating chronic inflammation, including ‘trapping’ MMPs and implementing effective sustained debridement, MMPs and biofilm production will be reduced [138].

The impairment of neutrophils also appears to be caused by quorum sensing (QS)-regulated proteases [139]. Active QS mechanisms associated with *P. aeruginosa* in immunocompromised patients with chronic infections [140] results in an accumulation of circulating neutrophils close to the biofilm [141]. Jensen et al. observed that chronic venous leg ulcers containing *P. aeruginosa* had a significantly higher number of neutrophils than wounds containing *S. aureus*, suggesting once again that biofilms are surrounded or overlaid by neutrophils, but they are not penetrated and killed by them [140]. Regarding macrophages, normally, they are activated by the presence of microorganisms and their by-products such as lipoteichoic acid (LTA), lipopolysaccharide (LPS) and chemical mediators [142]. Macrophage activation promotes the release of pro-inflammatory cytokines and cytotoxic molecules including tumour necrosis factor-α (TNF-α), interleukin (IL)-1, IL-6, IL-8 and nitric oxide (NO) [143]. The role of macrophages in chronic biofilm infection is still unclear. Some studies suggest that macrophages release more proinflammatory cytokines (TNF-a, IL-6) and NO levels when exposed to planktonic cells than to biofilms [142]. Therefore, chronic inflammatory response is not always successful in removing the biofilm [105]. Instead, it has been hypothesised that chronic inflammation may benefit the biofilm by inducing an ineffective inflammatory response [144].

#### 7.1.1. Clinical Profile

A limited number of studies correlate biofilm formation with DFUs. According to Zubair et al., 77.1% DFU patients had an infection of biofilm-producing bacteria, and the presence of a biofilm was associated with gender, neuropathy presence, osteomyelitis, ulcer duration, ulcer grade, necrotising ulcer and ulcer size [145]. Malik et al. determined that the overall biofilm-producing infection rate among DFUs was 67.9% and involved the same risk factors highlighted by Zubair et al. [145,146]. Vatan et al. found that the overall rate of biofilm production among 339 wound isolates was 34% [147]. Once again, the same risk factors for biofilm formation in DFU were observed, except for ulcer size, which was not assessed. Additionally, Pugazhendhi and Dorairaj demonstrated the correlation of biofilm formation with the clinical characteristics of DFU [148]. In this way, ulcer duration, size, nature and grade were associated with biofilm production. Therefore, biofilm formation in DFUs appears to be mainly linked with all the factors mentioned above (Table 3) [146,147,148].

Many of the bacterial species found in chronic wounds are commensals on the skin [41]. This transfer of microorganisms from healthy skin to the wound tissue can increase infection severity [108,109,110]. Biofilm formation in DFUs is more commonly associated with Gram-negative bacteria than those which are Gram-positive (Table 4). In the study by Banu et al., the rate of biofilm formation in DFU isolates was 46%, from which 26.5% were *P. aeruginosa* and 10.5% were *E. coli* strains [149]. Mottola et al. demonstrated that *Pseudomonas* strains had the highest rate of biofilm production, followed by *Corynebacterium, Acinetobacter, Staphylococcus* and *Enterococcus* strains [150]. Additionally, Vatan et al. verified that the most common biofilm-associated bacteria in DFUs were *A. baumannii* (65%*), P. aeruginosa* (52%) and *Klebsiella* spp. (40%) [147].

#### 7.1.2. Diagnosis and Treatment Using Conventional Methods

**Figure 1:**
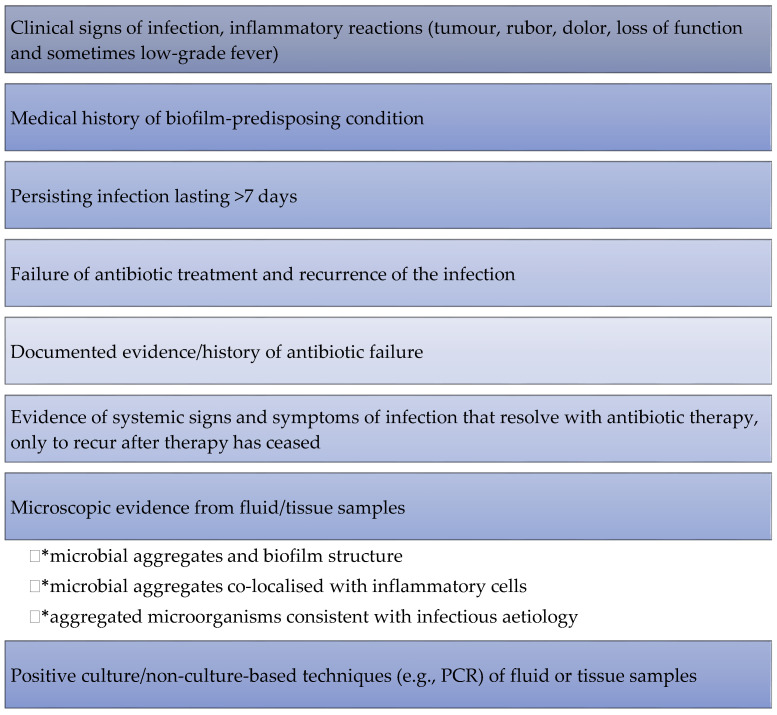
Clinical and laboratory indications for diagnosis of biofilm infections—ESCMID General Features. Based on Høiby et al. [124].

Contrary to planktonic microorganisms that can be easily identified through cultivation-based approaches, biofilm identification requires specific techniques. Magana et al. described current methods for bacterial biofilm characterisation, monitoring and quantification in detail [151]. However, some of these methods are not available in most clinical microbiology laboratories [152]. Additionally, there is an evident lack of tests for the detection of biofilms in wounds, as well as quantifiable biomarkers to confirm their presence. Consequently, the search for biofilms in clinical samples is difficult and time-consuming [124]. To work around this flaw, some clinicians use ‘clinical cues’ of the biofilm presence through naked eye observations. Such signs include the presence of a shiny, translucent and slimy layer on the non-healing wound surface; the presence of slough or fibrin; and the presence of gelatinous material that quickly returns after removal [104]. Figure 1 provides a list of the main clinical and laboratory indications for the diagnosis of biofilm infections according to the European Society of Clinical Microbiology and Infectious Diseases (ESCMID) guidelines.

Inaccurate sample collection can frequently lead to false-negative results [124]. Tissue biopsies are considered the most reliable type of sample to reveal biofilms in wounds [124]. The use of swabs for the collection of biofilm samples from the wound surface is not recommended [124]. In fact, contamination can occur from the skin flora, in addition to the strong adherence of biofilms to the epithelium and growth of anaerobes in deep tissues [80,118].

The detection of biofilms can be performed through routine light microscopy analysis and routine staining methods, including Gram staining, which stains tissues/mucus, inflammatory cells, bacteria and the biofilm matrix; and it requires evidence of an infectious process, such as the presence of leukocytes and microbial aggregates embedded in a self-produced matrix distinct from the surrounding tissue [153]. Confocal laser scanning microscopy (CLSM) and scanning electron microscopy (SEM) are the most appropriate techniques to reveal biofilms in biopsies, but they are not available in clinical settings for routine diagnostic work [124]. For instance, in the study performed by Johani et al., SEM and peptide nucleic acid fluorescent in situ hybridisation (PNA-FISH) techniques combined with CLSM were employed to analyse DFUs samples from 65 patients [104]. They identified the presence of densely aggregated bacteria often surrounded by an extracellular matrix in all samples, confirming that biofilms are ubiquitous in DFUs. Furthermore, the specific microscopic identification of biofilm microorganisms can also be performed by means of species-specific FISH probes and fluorescence microscopy [153]. In contrast, conventional culture methods or culture-independent methods based on PCR techniques (16S rRNA gene amplification, denaturant gradient gel electrophoresis, bacterial tag-encoded FLX amplicon pyrosequencing) [154,155] cannot discriminate between planktonic and biofilm-growing bacteria [156,157,158].

According to the current guidelines [81,159], the treatment of biofilm-associated DFUs infections should be based on a biofilm-based wound treatment (BBWC) that emphasises a “step-down-then-step-up” approach. This treatment line begins with a combination of the mechanical debridement of biofilms in an aggressive manner and the administration of topical antibiotics effective for killing residual bacteria from the biofilm. As soon as the biofilm bioburden level is reduced, the inflammatory response (neutrophils and macrophages) and the high levels of proteases and ROS will also decrease. This will allow the wound to evolve from a chronic state to an active healing state. At this point, the frequency of topical treatments can then “step down” but not stop completely. Finally, because the wound bed is “biofilm-free”, topical treatments can “step up” advanced wound treatments, which will effectively stimulate healing [152]. Here, we propose an algorithm for the detection and treatment of biofilms in DFUs (Figure S1) based in BBWC that emphasise a “step-down-then-step-up” [159,160,161].

#### 7.1.3. Unconventional Therapeutic Strategies

Biofilms have an important role in the aetiology and treatment failure of DFUs, which require new approaches to combat this kind of infections, particularly when MDR bacteria are involved [41]. To overcome this hurdle, alternative strategies and approaches to deal with biofilm-associated infections are being investigated. Unfortunately, there have been few studies that focused on the treatment of biofilm-associated DFUs. A search on SCOPUS (2021) with the words: “Antibiofilm diabetic foot ulcer” in the title, keywords and abstract only displayed eleven research items: three reviews [118,162,163]; seven research articles about new antibiofilm approaches [164,165,166,167,168,169,170]; and one about the design of antibiofilm peptides [171]. Thus, in this review, the most promising molecules and techniques to combat biofilm formation or to disaggregate already established mature biofilms, and that can be used in the prevention/control of biofilm-associated DFU infections, will be summarised (Table S1). They include antimicrobial peptides (AMPs), bacteriophages, phytochemicals, nanoparticles and photodynamic therapy (PDT).

AMPs represent a very promising approach as an alternative to antibiotics for treating chronic biofilm-based infections [172,173,174,175,176,177,178,179,180,181,182]. AMPs have the ability to interfere with various stages of the biofilm mode of growth, which can be related to their different mechanisms of action [183]. For example, peptide 6K-F17 can interact with the EPS produced by *P. aeruginosa*, leading to reductions in biofilm formation [184]. On the other hand, human cathelicidic LL-37 can prevent the biofilm formation of *P. aeruginosa* in three ways [185]. First, LL-37 reduces the initial cell attachment of *P. aeruginosa* to the surface, resulting in fewer bacteria involved in the early stages of biofilm development [185]. Secondly, LL-37 promotes twitching motility mediated by the type IV pili, by stimulating the expression of genes related to type IV pilus biosynthesis and function [185,186]. Increased surface motility can cause bacteria to wander across the surface instead of forming biofilms, resulting in thin and flat biofilms (without a mushroom-like structure) [187,188]. Thirdly, LL-37 affects Las and Rhl, the two major QS-systems of *P. aeruginosa*. Another example is the interference of some AMPs with the signalling nucleotides guanosine 5′-diphosphate 3′-diphosphate (ppGpp) and (p)ppGpp that regulate the expression of a plethora of genes [189,190], which, in turn, are important for biofilm formation [191]. The AMPs IDR-1018, DJK-5 and DJK-6 can block the synthesis and trigger the degradation of (p)ppGpp in both Gram-positive and Gram-negative bacteria, which may result in the reduction in biofilm formation [192,193,194]. Additionally, these peptides can act synergistically with some conventional antibiotics (e.g., ceftazidime, ciprofloxacin, imipenem and tobramycin) against Gram-negative pathogens, reducing the antibiotic concentrations required for complete biofilm inhibition up to 64-fold [192,195].

Due to ineffective antibiotic therapy to prevent and control biofilm infections, another strategy that has aroused interest is phage therapy [196,197,198,199,200,201]. Bacteriophages are naturally occurring viruses that can infect and kill bacteria [202]. Unlike many antibiotics, bacteriophages are able to target bacteria within biofilms without inducing resistance [203]. Bacteriophages can be lysogenic when they coexist with the host by inserting themselves into the bacterial genome, or lytic when they destroy the host by replication. Lytic bacteriophages are the most suited type for therapeutic use [203]. For example, bacteriophage ZCKP1 was tested in vitro to evaluate its lytic activity against an MDR *K. pneumoniae* KP/01, isolated from DFUs [204]. ZCKP1 bacteriophage had the ability to reduce the bacterial counts of host bacteria by ≥2 log_10_ CFU/mL at 25 °C, and consequently, the biofilm mass (>50%) [204]. Bacteriophages can be used individually or in a combination of multiple bacteriophages with different host ranges and directed to different receptors, the so-called bacteriophage cocktails. Bacteriophage cocktails allow a greater spectrum of activity and prevent the development of bacterial variants resistant to their action [205]. Alves et al. studied the prevention of *P. aeruginosa* PAO1 biofilm formation using a cocktail of six phages [206]. Two biofilm models were studied, one static and one dynamic, and the bacteriophage cocktail was assessed for its ability to reduce and disperse the biofilm mass. For the static model, more than 95% of biomass was eliminated after 4 h of contact with the bacteriophage suspension. In the flow biofilm model, a slower rate of activity was observed, but 48 h after addition of the bacteriophage cocktail the biofilm was dispersed, promoting a reduction of >4-log [206]. In addition, bacteriophages can also be combined with antibacterial agents, such as antibiotics, to improve the effectiveness against biofilms [201,207,208,209]. Rahman et al. combined the bacteriophage SAP-26 with three different antibiotics (rifampicin, azithromycin and vancomycin) for the control of *S. aureus* biofilms [207]. The best result was observed for the combination SAP-26/rifampicin after 24 h of treatment, disrupting the biofilm matrix and promoting a 4-log reduction [207]. Chaudhry et al. tested the combination of two bacteriophages (NP1 and NP3) with antibiotics from five distinct classes (ceftazidime, ciprofloxacin, colistin, gentamicin and tobramycin) against *P. aeruginosa* biofilms [208]. Biofilms were treated in one of two ways: the bacteriophages and antibiotics were simultaneously added; or the bacteriophage mixture was added first and then antibiotics were added with a delay of 4 or 24 h. A significant effect was observed when bacteriophages were added first and only after 24 h, when gentamicin or tobramycin were added (1.5-log and 2-log reductions, respectively) [208]. Similarly, Akturk et al. evaluated the synergistic activity of the bacteriophage EPA1 in combination (simultaneous and sequential) with different antibiotics (gentamicin, kanamycin, tetracycline, chloramphenicol, erythromycin, ciprofloxacin, and meropenem) against mono and dual *P. aeruginosa* and *S. aureus* biofilms [209]. These authors found an improvement in the killing effect when EPA1 and antibiotics were applied simultaneously. Moreover, a biofilm reduction below the detection limit was observed when gentamicin or ciprofloxacin were added sequentially after 6 h of bacteriophage treatment. For dual-species biofilms, increasing the gentamicin concentration was needed to obtain a similar killing effect as in mono-species [209].

Phytochemicals (molecules from the secondary metabolism of plants) are considered a green and sustainable source of effective antibiofilm molecules [210]. The use of phytochemicals for the control of multi-resistant biofilms has already been extensively reviewed [210,211,212,213,214,215,216]. Due to their great structural diversity, phytochemicals are associated with a multi-target mode of action, without imposing selective pressure on bacteria [210]. Borges et al. observed that ferulic acid and gallic acid were effective in the prevention and control of biofilms of *E. coli, Listeria monocytogenes, P. aeruginosa* and *S. aureus* [217]. Monte et al. investigated the mode of action of 7-hydroxycoumarin and indole-3-carbinol against *E. coli* and *S. aureus* biofilms, and observed that both phytochemicals affected the motility and QS activity [218]. Ouyang et al. observed a significant inhibition of biofilm formation and production of virulence factors, including pyocyanin, protease and elastase in *P. aeruginosa* by quercetin [219]. In addition, they detected transcriptional changes associated with QS and observed that the expression levels of *lasI*, *lasR*, *rhlI* and *rhlR* were reduced [219].

Nanoparticles (NPs) have been extensively examined as drug delivery systems for the treatment of wound infections [220,221]. Due to the biofilm EPS and its ability to hijack, inactivate, or inhibit the action of antibiotics, there is a need to design drug delivery systems for the treatment of chronic infections [222]. A delivery system will make it possible to “protect” the drug, allowing the release of the drugs in a controlled manner [223,224,225]. NPs are usually classified into metallic NPs, nonmetal NPs, polymeric NPs, lipid NPs, quantum dots, and ceramic NPs. This review focuses only on one type of metallic NPs, silver NPs (AgNPs), because their antimicrobial efficacy and potential for use in the treatment of chronic infections has been extensively reported [226,227]. Martínez-Gutierrez et al. evaluated the antibiofilm activities of AgNPs against *P. aeruginosa* PAO1 biofilms generated under static conditions and high fluid shear conditions [228]. They observed log reductions inversely related to fluid shear: for biofilms formed under fluid shear, inhibition by AgNPs occurred at a concentration of 100 mg/mL resulting in a 4-log reduction; for a 4-log reduction in biofilms under static conditions, a concentration of 500 mg/mL was required. This difference shows that the formation of biofilms under shear forces can change their structure and permeability. More recently, in a study performed by Appapalam et al., the antibiofilm action of aqueous extracts of *Aerva lanata* silver nanoparticles (AL-AgNPs) against the most predominant and antibiotic-resistant DFU bacterial isolates (*P. aeruginosa, E. coli, S. aureus* and *Bacillus subtilis*) was efficiently observed [229]. The minimum inhibitory concentration (MIC) and minimum bactericidal concentration (MBC) of AL-AgNPs against the DFU isolates were 5–15 mg/mL and 10–20 mg/mL, respectively. MIC and MBC of AL-AgNPs were effective in destroying the preformed biofilms of DFU isolates [229]. Furthermore, AL-AgNPs at MBC displayed an increased intracellular accumulation of reactive oxygen species (ROS), membrane leakage, permeability, cell damage and genotoxicity in the DFU isolates, which suggests an action on different targets and through different modes of action [229].

Physical methods (e.g., irradiation, heat, high pressure) have also gained popularity for their potential to induce resistance and are amenable for large scale applications [230]. Among these methods is PDT, which consists of the application of photosensitisers to enhance the activity and transport of the antibiotics through of the biofilms [223,224]. At a clinical level, PDT involves the topical application of a photosensitiser into the tissue, followed by illumination to induce the formation of ROS [231]. PDT provides bacterial inactivation and promotes wound healing, and can be used to manage the infection and microbial colonisation of DFUs [232]. Recently, some studies have emerged on the PDT approach to combat microbial biofilms [36,233,234], including the combination of PDT with antibiotics [36,234,235]. For instance, Barra et al. showed that an earlier treatment of a biofilm formed by Gram-positive bacteria (*S. aureus* and *S. epidermidis)* with 5-aminolevulinic acid (5-ALA)/PDT increased the susceptibility to gentamicin [234]. The authors concluded that the ROS-mediated destruction, or significant damage, of the biofilm may have enhanced the penetration and the subsequent action of the antibiotic [234]. The effects of PDT on bacterial viability within biofilms were evaluated after pre-incubation with 5-ALA and irradiation with light fluences from 25 to 500 J/cm^2^. The viability of all types of cells analysed decreased with the increase in the light dose administered [234]. Similarly, Li et al. exposed biofilms of *S. aureus* and MDR *P. aeruginosa* to indocyanine green (ICG) photosensitiser and ethylenediamine tetraacetate (EDTA) metal chelating agent, alone or combined with antibiotics (vancomycin and amikacin) followed by irradiation [34]. They observed that PDT-ICG + EDTA induced more bacterial death than PDT-ICG. After treatment with PDT-ICG + EDTA followed by amikacin, biofilm detachment increased and bacterial viability decreased. When PDT-ICG + EDTA-treated biofilms were combined with vancomycin, the integrity of the cellular membranes was compromised, resulting in a small percentage of living cells [36]. These results can be justified by the fact that EDTA counteracts biofilms by chelating Mg^2+^ and Ca^2+^, which leads to cell wall instability and the increased permeability of antibiotics and/or photosensitisers [236,237,238]; furthermore, EDTA also removes iron atoms, which are essential for microbial virulence and pathogenicity [238].

## 8. Concluding Remarks and Challenges

People with diabetes have increased challenges in the wound healing process, which puts them at a higher risk of developing chronic ulcers. DFUs have a significant impact on the morbidity and mortality of patients as well as from an economic standpoint. This impact is even greater when biofilms are present. Biofilms are not a new concept but continue to be frequently undervalued despite their prominent role in the chronicity of diabetic wounds. Biofilm-associated DFUs are usually treated as a simple infection, consisting of basic wound care and the administration of current antibiotics, which is often insufficient. Equally worrying is the lack of implementation of biofilm infection diagnosis tools. The clinical outcomes for patients with DFUs become much more aggravated when biofilms are present; therefore, the implementation of diagnostic techniques directed for identifying biofilms in routine clinical practices is strongly suggested.

In recent years, some guidelines have emerged to direct the diagnosis and treatment of biofilm infections. According to these guidelines, the treatment of biofilm-associated DFU infections should be based on the principles of BBWC, with a combination of debridement and the administration of antibiotics. Additionally, the use of algorithms for the detection and treatment of biofilm-associated DFUs can be useful in clinical practice. To the best of our knowledge, there are no algorithms that consider detection and treatment simultaneously. Here, we proposed an algorithm that combines both to enable the detection of DFUs and assist in choosing the treatment line to follow. However, when MDR bacteria are involved, new strategies capable of inhibiting biofilm formation or dispersing preformed biofilms, without conferring selective pressure, are required.

Despite the efforts, biofilm-associated DFUs are a problem which are still far from being solved. Diabetes alone leads to dysfunctions in wound healing; therefore, it is not accurate to affirm that if the biofilm is treated, the wound will heal. That said, better evidence about the role of biofilms in DFU healing is necessary. Only in that way can new treatment options emerge and translate into the clinical practice.

## Figures and Tables

**Table 1:** Suggested antibiotics for the empirical treatment of infected DFUs based on the clinical severity. Adapted from Lipsky et al. [80].

Severity	Associated Pathogen(s)	Additional Factor(s)	Antibiotic(s)
Mild (topical or oral antibacterial agents)	*Staphylococcus aureus* (MSSA) *Streptococcus* spp.	No complicating features	First-generation cephalosporin, nafcillin, ampicillin/sulbactam, amoxicillin/clavulanate, clindamycin
		β-lactam allergy or intolerance	Clindamycin, levofloxacin, moxifloxacin, doxycycline
		Recent antibiotic exposure	Levofloxacin, moxifloxacin, second- or third-generation cephalosporin
	MRSA		Clindamycin, doxycycline, trimethoprim/sulfamethoxazole
Moderate (oral or initial parenteral antibacterial agents) or Severe (parenteral antibacterial agents)	MSSA *Streptococcus* spp. Enterobacteriaceae obligate anaerobes	No complicating features	Second- or third-generation cephalosporin, aminoglycoside
		Recent antibiotic exposure	Third-generation cephalosporin, aminoglycoside, ertapenem, piperacillin/tazobactam, cefepime
	*Pseudomonas aeruginosa*		Piperacillin/tazobactam, cefepime, imipenem, meropenem
	MRSA Enterobacteriaceae obligate anaerobes		Vancomycinc plus one of the following: ceftazidime, cefepime, piperacillin/tazobactam, aztreonam, or a carbapenem
	ESBL, MDR Gram-negative		Piperacillin/tazobactam plus one of the following: aminoglycoside, or a carbapenem

**Table 2:** Classes of dressings used in DFUs. Adapted from Hilton et al. [85] and Kavitha et al. [88].

Dressing Classes	Advantage(s)	Disadvantage(s)
Tulle	Good, moist environment	Be careful not to dry
Low-adherence	Hypoallergenic; Inexpensive; Moist environment	Minimal absorbency
Polyurethane films	Water-proof dressing; Comfortable; Transparent (allows wound monitoring)	Facilitates maceration
Hydrocolloids	Absorbent; Can be left for several days; Aids autolysis	Avoid use on infected wounds; Facilitates maceration; Unpleasant odour
Hydrogels	Good absorbent; Aids autolysis; Donate liquid	Avoid use on infected wounds; Facilitates maceration
Foams	Thermal insulation; Good absorbent	Can adhere to wound; Occasional dermatitis due to the adhesive
Alginates	Highly absorbent; Bacteriostatic; Haemostatic; Useful in cavities	Require wetting before removal
Iodine preparations	Antiseptic; Moderately absorbent	Iodine allergy; Discolours wounds; Avoid in cases of thyroid disease or pregnancy
Silver-impregnated	Antiseptic; Absorbent	Cost

**Table 3:** Relationship between biofilm formation and clinical profile. Comparison of 4 studies (Zubair et al. [145]; Malik, Mohammad and Ahmad [146]; Pugazhendhi and Dorairaj [148]; Vatan et al. [147]).

	Study 1	Study 2	Study 3	Study 4
*n* *	57	162	-	160
**Biofilm +**	44	110	115	-
**Gender** (male)	32	76	-	82
**Age distribution**	44.6 ± 7.3	-	62.7	-
>40 years	64.8%	70.0%	-	-
**Diabetes duration** (average year)	14.9 ± 2.6	-	16.0 ± 0.0	-
**Ulcer duration**	39.6 ± 2.6	-	48.2 ± 42.3	-
>1 month	52.6%	75.0%	-	
**Hospital stay**				
>1 month	59.5%	75.0%	-	-
**Amputation**	24.5%	80.4%	42.0%	
**Ulcer size**				
<4 cm^2^	64.9%	69.3%	-	89.0%
**Comorbidities**				
Hypertension	72.7%	80.4%	34.0%	16.0%
Nephropathy	77.1%	77.7%	37.0%	25.0%
Retinopathy	68.7%	52.4%	3.0%	22.0%
Neuropathy	89.4%	57.3%	34.0%	18.0%
Osteomyelitis	88.8%	65.0%	-	-
**Status**				
Death	3.5%	72.2%	38.0%	-

* The *n* value represents the number of diabetic patients with foot ulcers, infected or not infected with biofilms.

**Table 4:** The main biofilm-producing bacteria isolated from DFUs.

	Reference
**Gram-negative**	
Enterobacteriaceae	[147]
*Escherichia coli*	[145,146,149,151,152]
*Klebsiella* spp.	[152]
*Klebsiella pneumoniae*	[145,146,151]
*Klebsiella oxytoca*	[145,146,149]
*Pseudomonas* spp.	[150]
*Pseudomonas aeruginosa*	[145,146,147,149,151,152]
*Proteus* spp.	[149]
*Proteus vulgaris*	[145,146]
*Proteus mirabilis*	[146]
*Acinetobacter* spp.	[145,146,150,151]
*Acinetobacter baumani*	[147]
*Morganella morganii*	[145,146]
*Vibrio* spp.	[152]
*Citrobacter* spp.	[149,151]
**Gram-positive**	
*Coryneform* spp.	[146]
*Corynebacterium* spp.	[150]
Beta-haemolytic *Streptococcus*	[146,151]
Coagulase-negative *Staphylococcus* spp.	[146]
*Staphylococcus* spp.	[150,152]
*Staphylococcus aureus*	[146,147,149,151]
MRSA	[149]
*Enterococcus* spp.	[147,150]
*Enterococcus faecalis*	[146]

## Supplementary Materials

The following are available online at https://www.mdpi.com/article/10.3390/ijms22158278/s1, Figure S1: Proposed “step-down-then-step-up” approach in the detection and treatment of biofilms in DFUs (based on Metcalf et al. [160], Percival et al. [161] and Schultz et al. [159]); Table S1: Emerging therapeutic approaches against the main biofilm-forming pathogens commonly associated with DFUs infections.
