# Supplementary material for: Biofilms in Diabetic Foot Ulcers: Impact, Risk Factors and Control Strategies
Source: Int J Mol Sci. 2021 Jul 31;22(15):8278. doi: 10.3390/ijms22158278 (PMC8347492; doi:10.3390/ijms22158278)
Supplement: Supplementary file 1 [file ijms-22-08278-s001.zip › ijms-1279738-supplementary.pdf]

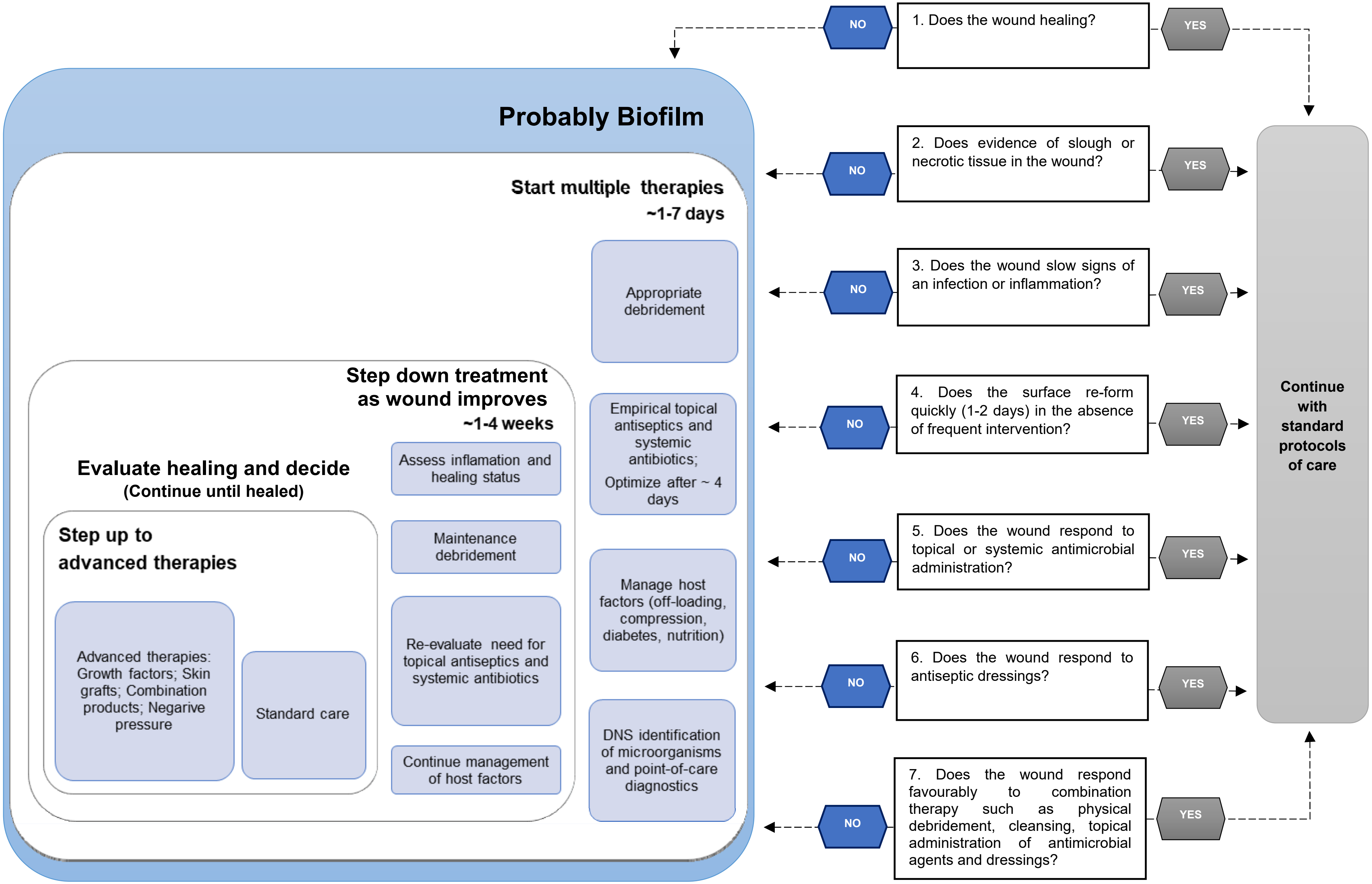
1. Does the wound healing?
2. Does evidence of slough or necrotic tissue in the wound?
3. Does the wound slow signs of an infection or inflammation?
4. Does the surface re-form quickly (1-2 days) in the absence of frequent intervention?
5. Does the wound respond to topical or systemic antimicrobial administration?
6. Does the wound respond to antiseptic dressings?
7. Does the wound respond favourably to combination therapy such as physical debridement, cleansing, topical administration of antimicrobial agents and dressings?
NO
YES
Continue with standard protocols of care
Probably Biofilm
Start multiple therapies ~1-7 days
Appropriate debridement
Empirical topical antiseptics and systemic antibiotics; Optimize after ~ 4 days
Manage host factors (off-loading, compression, diabetes, nutrition)
DNS identification of microorganisms and point-of-care diagnostics
Step down treatment as wound improves ~1-4 weeks
Assess inflamation and healing status
Maintenance debridement
Re-evaluate need for topical antiseptics and systemic antibiotics
Continue management of host factors
Evaluate healing and decide
(Continue until healed)
Step up to advanced therapies
Advanced therapies: Growth factors; Skin grafts; Combination products; Negarive pressure
Standard care

Approach		Molecular structure	Biofilm-forming pathogens	Source	Mechanism(s)	Dose/Effect	Reference(s)
Antimicrobial peptides	Pexiganan	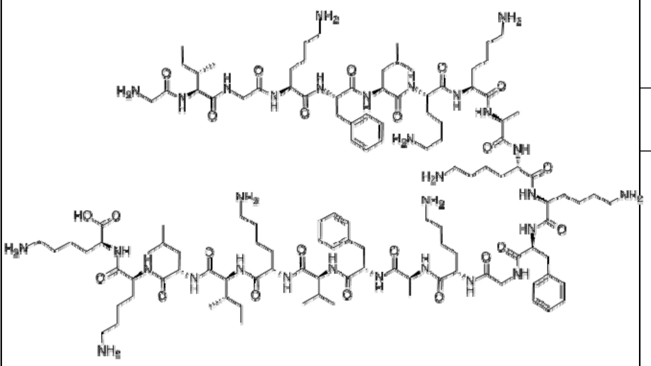	*P. aeruginosa* Z25.1	DFU	n.d.	MBIC = 80 μg/ml MBEC = 100 μg/ml	[182]
			S. aureus Z25.2	DFU	n.d.	MBIC = 10 μg/ml MBEC = 9 μg/ml	[182]
			P. aeruginosa + *S. aureus*	DFU	n.d.	MBIC = 50 μg/ml MBEC = 120 μg/ml	[182]
	Pexiganan + Nisin	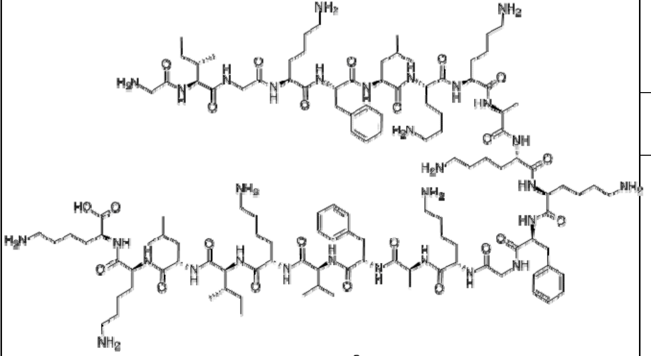 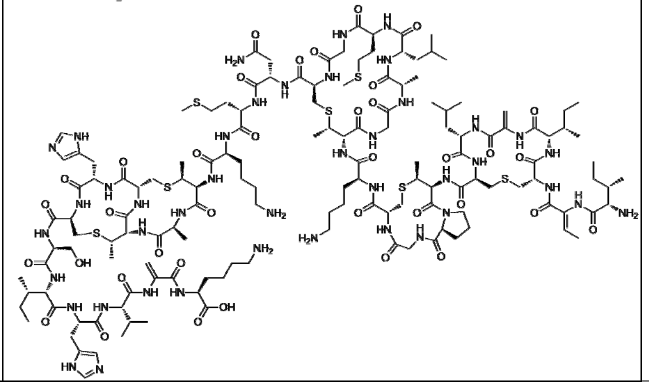	*P. aeruginosa* Z25.1	DFU	n.d.	MBIC = 70 MBEC = 90 μg/ml	[182]
			S. aureus Z25.2	DFU	n.d.	MBIC = 1 μg/ml MBEC = 5 μg/ml	[182]
			P. aeruginosa + *S. aureus*	DFU	n.d.	MBIC = 50 μg/ml MBEC = 50 μg/ml	[182]

	6K-F17	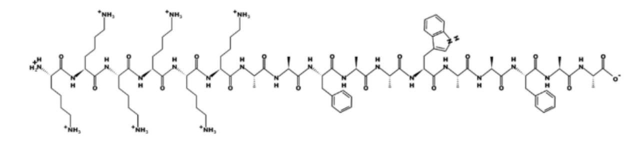	*P. aeruginosa* PAO1	Reference strain	Interaction with biofilm EPS	10 µg/ml (75% biofilm reduction)	[184]
	SAAP-148	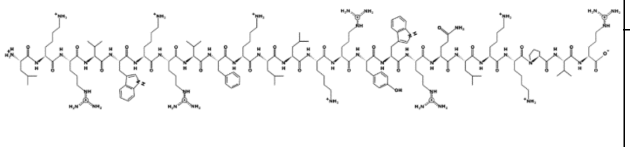	*A. baumannii*	Clinical isolates	Interaction and subsequently permeabilization of the cytoplasmic membrane, leading to bacterial death	12.8 mM (85% biofilm reduction)	[177]
			S. aureus	Clinical isolates		12.8 mM (81% biofilm reduction)	
	Magainin 2	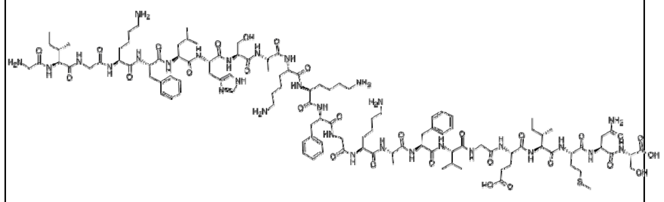	*A. baumannii*	Clinical isolates	n.d.	256 µM (66% biofilm inhibition)	[178]
	gH625	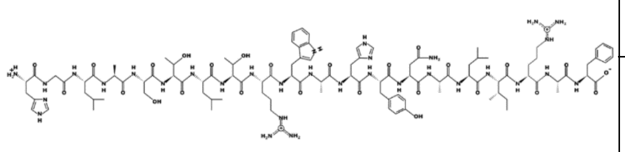	*S. aureus*	Otitis infection	Biofilm penetration and interaction with microbial membranes. The modification of the gH625 peptide, which consists of the addition of a sequence of lysine residues, increased the capacity of penetration and interaction	50 µM (35% biofilm prevention)	[179]
			Polimicrobial biofilms (*Candida tropicalis* and *S. aureus*)	Systemic infection and otitis infection		50 µM (48% biofilm prevention)	
	gH625- GCGKKKK	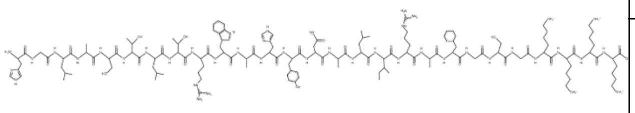	*S. aureus*	Otitis infection		50 µM (50% biofilm prevention)	
			Polimicrobial biofilms (*C. tropicalis* and *S. aureus*)	Systemic infection and otitis infection		50 µM (88% biofilm prevention)	

	Hp1404-T1e	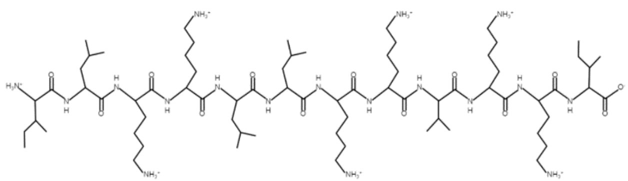	*P. aeruginosa*	ATCC 27853	n.d.	MBIC = 12,5 μM	[180]
	DJK-5	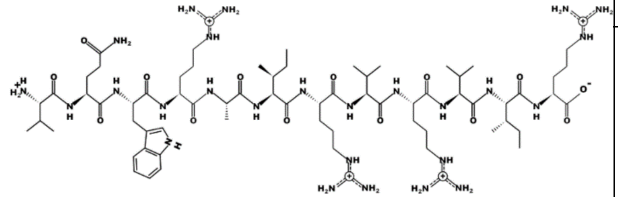	*P. aeruginosa*	Cystic fribrosis	Binding and promotion of degradation of the signal for biofilm formation and maintenance (p)ppGpp	$MBIC_{50}$ = 1 µg/ml	[192]
			K. pneumoniae	Clinical isolates		$MBIC_{50}$: 8 to >64 µg/ml	[194]
	DJK-6	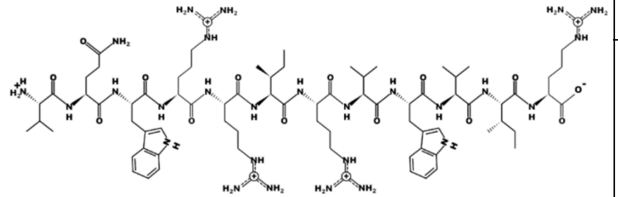	*K. pneumoniae*	Clinical isolates		$MBIC_{50}$: 1 to 8 µg/ml	
			P. aeruginosa	Cystic fribrosis		$MBIC_{50}$=0,5µg/ml	[192]
	FI-$P_RP_RP_L$-5	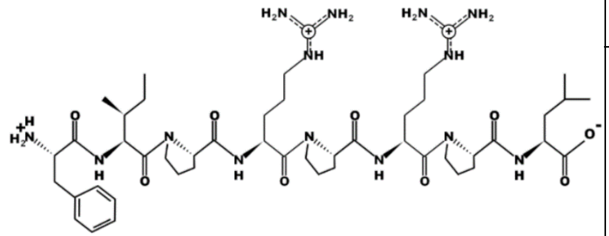	*S. epidermidis*	-	n.d.	40 % biofilm reduction	[173]
			S. aureus	-		49% biofilm reduction	
	IDR-1018	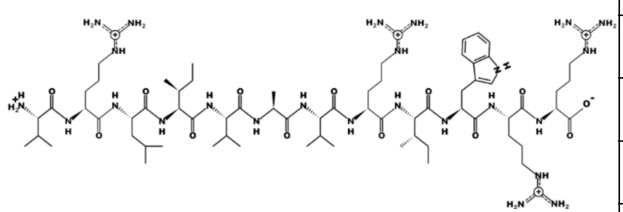	*A. baumannii*	Clinical isolates	Binding and promotion of degradation of the signal for biofilm formation and maintenance (p)ppGpp	$MBIC_{50}$ = 2 µg/ml	[193]
			E. coli			$MBIC_{50}$ = 8 µg/ml	
			K. pneumoniae	ATTC 13883		$MBIC_{50}$ = 2 µg/ml	
			MRSA	Clinical isolates		$MBIC_{50}$ = 2 µg/ml	
			P. aeruginosa PAO1	Wild type		$MBIC_{50}$ = 5 µg/ml	

	1037	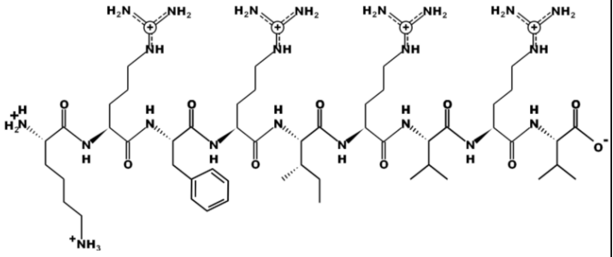	*P. aeruginosa* PAO1	Reference strain	Inhibition of swimming and swarming motilities and stimulation of twitching motility	10 µg/ml (50% biofilm inhibition)	[186]
	LL-37	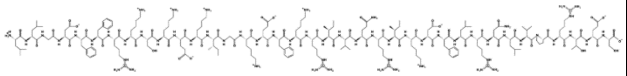	*S. aureus*	ATCC 25923	Affect the expression of *agr* locus and *ica* locus genes, essential for the development of biofilm	10 µg/ml (40% biofilm inhibition)	[172]
			P. aeruginosa PAO1	Reference strain	Decrease attachment, increase twitching motility and affect *Las* and *Rhl* QS systems	16 µg/ml (75% biofilm reduction)	[185]
**Bacteriophages**	vB_PaeM_LS1		*P. aeruginosa*	Human isolates	Biofilm bacteria are susceptible to lytic bacteriophages	98% biofilm reduction (from 7.3 to 4.8 log_{10} CFU/ml)	[200]
	ZCKP1		*K. pneumoniae*	DFU		$\approx 60 - 90$% biofilm viability after 24h, single treatment	[204]
	NP1 + NP3 + Gentamicin	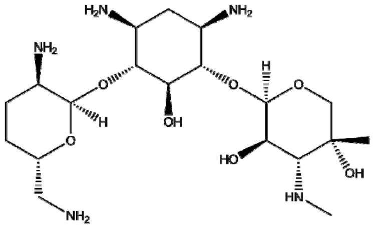	*P. aeruginosa*	-		1.5-log reduction	[208]

	NP1 + NP3 + Tobramycin	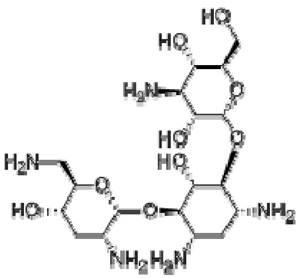	*P. aeruginosa*	-		2-log reduction	[208]
	vB_AbaM-IME-AB2		*A. baumannii*	Expectoration		88.5% biofilm inhibition (5.6 × 10^{-7} CFU)	[199]
	Bacteriophage cocktail (DL52, DL54, DL60, DL62, DL64 and DL68)		*P. aeruginosa* PAO1	Reference strain		95% biofilm reduction after 4h	[206]
	DRA88 + phage K		*S. aureus*	Clinical isolates		≈ 100% biofilm disruption after 48h	[197]
	Bacteriophage cocktail (*S. aureus* F44/10 and F125/10; *P. aeruginosa* F770/05 and F510/08; *A. baumannii* F1245/05)		*A. baumannii*	Clinical isolates		≈ 60% biofilm reduction after 24h, one-time bacteriophage application	[198]
			P. aeruginosa	Clinical isolates		≈ 50% biofilm reduction after 24h, one-time bacteriophage application	
			S. aureus	Clinical isolates		≈ 50% biofilm reduction after 24h, one-time bacteriophage application	
	AB7-IBB2		*A. baumannii*	Clinical isolates		80% biofilm reduction	[196]
	SAP-26		*S. aureus*	Clinical isolates		2-log reduction	[207]

	SAP-26 + Rifampicin	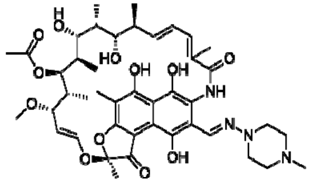	*S. aureus*	Clinical isolates		4-log reduction	[207]
**Phytochemicals**	α-mangostin	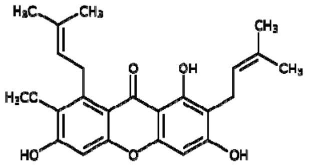	*P. aeruginosa*	DFU	n.d.	2 µg/ml	[169]
			S. epidermidis	DFU	n.d.	2 µg/ml	[169]
	Lutein	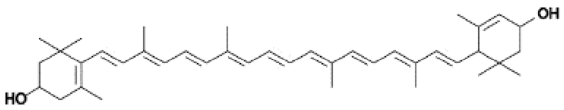	*P. aeruginosa* PAO1	Referenc e strain	n.d.	20 µg/ml (61% biofilm inhibition)	[167]
	Quercetin	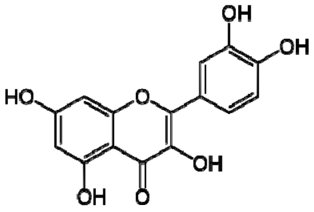	*P. aeruginosa* PAO1	Referenc e strain	Transcriptional changes associated with QS and expression levels of *lasI*, *lasR, rhlI* and *rhlR* significantly reduced	250 µg/ml (43 – 78% biofilm inhibition)	[213]
			P. aeruginosa PAO1	Referenc e strain		16 µg/ml (51% biofilm inhibition)	[219]
	Caffeic acid	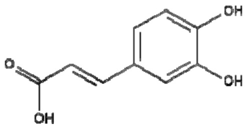	MRSA	clinical isolates	Interference with bacterial motility, changes in the physico-chemical properties of cell surfaces which alter the adhesion potential to PS, and interference with QS; and hinder the biofilm formation process	24% biofilm reduction	[212]
	p-coumaric acid	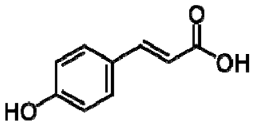				28% biofilm reduction	[212]
	Trans-cinnamaldehyde	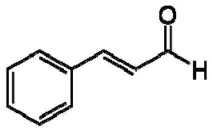				42% biofilm reduction; 79% inactivation	[212]

	7-hydroxycoumarin	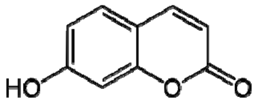	*E. coli*	CETC 434		800 µg/ml (51% biofilm reduction; 72% biofilm inactivation)	[217]
			S. aureus	CETC 976		200 µg/ml (10% biofilm reduction; 40% biofilm inactivation)	[217]
	Indole-3-carbinol	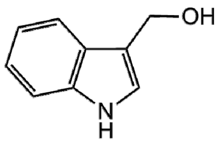	*E. coli*	CETC 434		800 µg/ml (43% biofilm reduction; 75% biofilm inactivation)	[217]
			S. aureus	CETC 976		400 µg/ml (47% biofilm inactivation)	[217]
	Salicylic acid	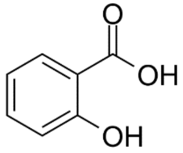	*E. coli*	CETC 434		3200 µg/ml (34% biofilm reduction; 75% biofilm inactivation)	[217]
			S. aureus	CETC 976		1600 µg/ml (47% biofilm inactivation)	[217]
	Saponin	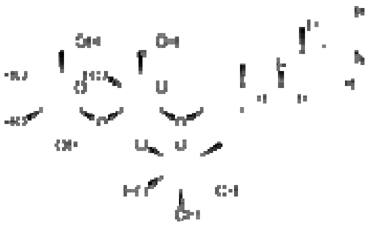	*E. coli*	CETC 434		3200 µg/ml (34% biofilm reduction; 75% biofilm inactivation)	[217]
			S. aureus	CETC 976		3200 µg/ml (48% biofilm inactivation)	[217]
	Allyl isothiocyanate	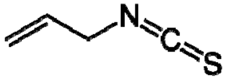	*E. coli*	CETC 434		1000 µg/ml (87% biofilm prevention)	[210]
			P. aeruginosa	ATCC 10145		1000 µg/ml (99% biofilm prevention)	[210]
			S. aureus	CETC 976		1000 µg/ml (96% biofilm prevention)	[210]

	2-phenylethyl isothiocyanate	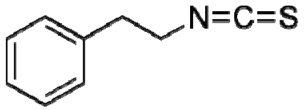	*E. coli*	CETC 434		1000 µg/ml (100% biofilm prevention)	[210]
			P. aeruginosa	ATCC 10145		1000 µg/ml (93% biofilm prevention)	[210]
			S. aureus	CETC 976		1000 µg/ml (90% biofilm prevention)	[210]
	Ferulic acid	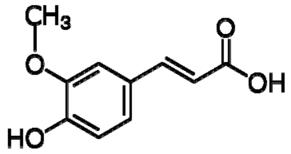	MRSA	Clinical isolates		27% biofilm reduction	[212]
			E. coli	CETC 434		2500 µg/ml (13% biofilm prevention; 38% biofilm reduction)	[216]
			P. aeruginosa	ATCC 10145		500 µg/ml (81% biofilm prevention; 42% biofilm reduction)	[216]
			S. aureus	CETC 976		5000 µg/ml (6% biofilm prevention)	[216]
	Gallic acid	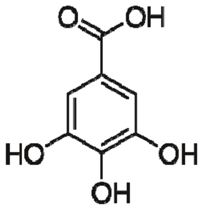	*E. coli*	CETC 434		5000 µg/ml (64% biofilm prevention; 29% biofilm reduction)	[216]
			P. aeruginosa	ATCC 10145		500 µg/ml (84% biofilm prevention; 48% biofilm reduction)	[216]
			S. aureus	CETC 976		500 µg/ml (70% biofilm prevention)	[216]
**Phytosynthesized silver nanoparticles (AgNPs)**	*Bridelia retusa* mediated AgNPs (BR-AgNPs)		*E. coli*	MTCC 739	Interference with QS	$MBIC_{50}$ = 64 µg/ml	[224]
			P. aeruginosa	MTCC 741		$MBIC_{50}$ = 23 µg/ml	[224]
			S. aureus	MTCC 96		$MBIC_{50}$ = 33 µg/ml	[224]

	Glochidion lanceolarium mediated AgNPs (GL-AgNPs)		*E. coli*	MTCC 739		$MBIC_{50}$ = 46 µg/ml	[224]
			P. aeruginosa	MTCC 741		$MBIC_{50}$ = 69 µg/ml	[224]
			S. aureus	MTCC 96		$MBIC_{50}$ = 53 µg/ml	[224]
	Semecarpus anacardium mediated AgNPs (SA-AgNPs)		*E. coli*	MTCC 739		$MBIC_{50}$ = 23 µg/ml	[224]
			P. aeruginosa	MTCC 741		$MBIC_{50}$ = 13 µg/ml	[224]
			S. aureus	MTCC 96		$MBIC_{50}$ = 34 µg/ml	[224]
	Aerva lanata mediated AgNPs (AL-AgNPs)		*B. subtilis*	DFU	n.d.	MIC (10 µg/ml; 35% biofilm disruption) MBC (15 µg/ml; 89% biofilm disruption)	[229]
			E. coli			MIC (5 µg/ml; 79% biofilm disruption) MBC (10 µg/ml; 99% biofilm disruption)	[229]
			P. aeruginosa			MIC (10 µg/ml; 45% biofilm disruption) MBC (15 µg/ml; 82% biofilm disruption)	[229]
			S. aureus			MIC (15 µg/ml; 47% biofilm disruption) MBC (20 µg/ml; 86% biofilm disruption)	[229]
	Piper betle mediated AgNPs (Pb-AgNPs)		*P. aeruginosa* PAO1	Referenc e strain	Inactivation of transcriptional regulator *lasR* and inhibition of AHL production	8 µg/ml (78 % biofilm inhibition)	[223]

	Gallic acid mediated AgNPS (GA-AgNPs)		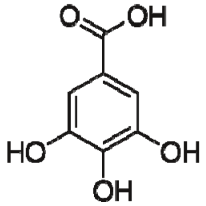	*P. aeruginosa* PAO1	Reference strain	n.d.	biofilm under high fluid shears conditions (100 mg/ml; ≈ 4-log reduction)	[228]
							biofilm under static conditions (500 mg/ml; ≈ 4-log reduction)	[228]
**Photodynamic therapy**	PDT-ICG		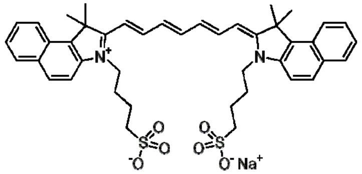	MDR *P. aeruginosa*	Clinical isolates	PDT generate a large amount of ROS, which then acted on bacterial cell walls, membranes and nucleic acids EDTA counteracts biofilms by chelating $Mg^{2+}$and Ca^{2+}; remove iron atoms that are essential for virulence and pathogenicity	MBIC (16 µg/ml; 90% survival ratio)	[36]
				MRSA			MBIC (32 µg/ml; 61% survival ratio)	[36]
	PDT-ICG + EDTA		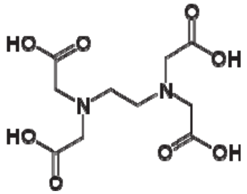	MDR *P. aeruginosa*			MBIC (16 µg/ml; 53% survival ratio)	[36]
				MRSA			MBIC (32 µg/ml; 55% survival ratio)	[36]
	PDT-ICG + EDTA	Amikacin	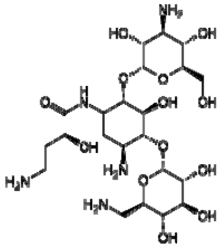	MDR *P. aeruginosa*			MBIC (16 µg/ml; 12% survival ratio)	[36]

		Vancomycin	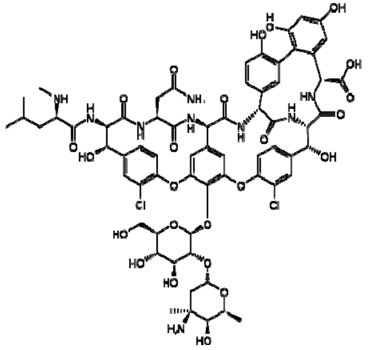	MRSA			MBIC (32 µg/ml; 13% survival ratio)	[36]
	PDT-5-ALA		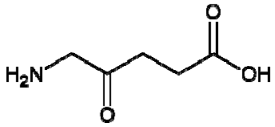	*S. aureus*	Clinical isolates	PDT generate a large amount of ROS, which then acted on bacterial cell walls, membranes and nucleic acids	500 J/cm^2 (20% survival ratio)	[234]
				S. epidermidis			500 J/cm^2 (20% survival ratio)	[234]
	PDT-5-ALA	Gentamicin	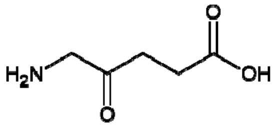 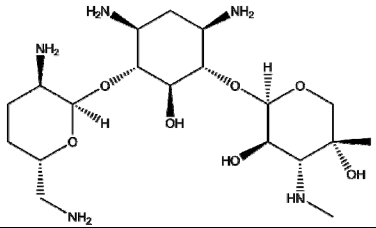	*S. aureus*			500 J/cm^2 + 2 µg/ml (≈ 15% survival ratio)	[234]
				S. epidermidis			500 J/cm^2 + 2 µg/ml (≈ 10% survival ratio)	[234]

	PDT-TMP	Vancomycin	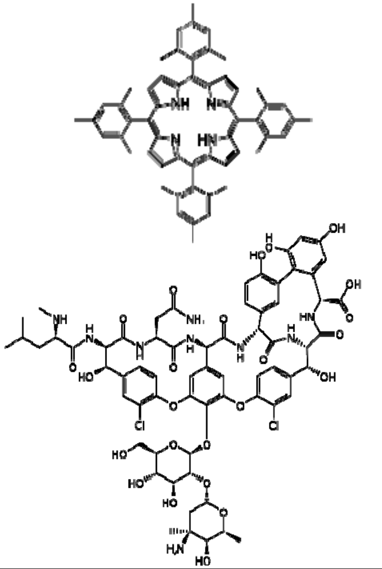	MRSA	Clinical isolates	EPS disruption	150 J/cm^2 + 10 μM TMP + 1 μg/ml	[235]

Minimal biofilm inhibitory concentration (MBIC); Minimal biofilm eradication concentration (MBEC); Minimal Biofilm Inhibitory Concentration leading to 50% decrease in biofilm growth (MBIC50); n.d. – not determined; American Type Culture Collection (ATCC); Spanish Type Culture Collection (CETC); Microbial Type Culture Collection (MTCC); Tetramesitylporphyrin (TMP)
